# Hospital Officials' Pensions

**Published:** 1922-04

**Authors:** 


*To the Editor of* The Hospital and Health Review.

*To the Editor of* The Hospital and Health Review.

### Hospital Officials' Pensions.

*To the Editor of* The Hospital and Health Review.

Sir,—We are under an obligation to you for the article which you have published in The Hospital and Health Review in regard to pensions for hospital officials. It is indeed strange that the permanent hospital employee should be one of the last to be thought of as worthy of consideration when his time of working is over, and, stranger still, that the employee in the medical schools attached to the hospitals should be enjoying the advantages of a pension scheme which up to the present are denied to others who work in the same sphere. Doubtless it is a matter which has been more or less overlooked, and there is no one to champion the cause of the hospital official. The secretariat cannot very well run a campaign of this kind—it is so obviously self-interested. One of the causes that has brought the medical school officials into the Federated Superannuation system for universities appears to be that the Board of Education has looked after the interests of the school teacher, and has obtained from the Treasury the non-recurrent grant which has benefited so materially the older employees in the schools. Although King Edward's Hospital Fund may not be able to obtain a non-recurrent grant to help the old hospital employee, it is most gratifying to hear that that fund is taking up a similar position in regard to the hospital employee to that adopted by the Board of Education in regard to the school teacher.

Yours, &c.,
A London Hospital Secretary.

